# New Diagnostic Score for Sepsis in Adult Horses with Acute Gastrointestinal Disease

**DOI:** 10.3390/ani16060962

**Published:** 2026-03-19

**Authors:** Angélique Blangy-Letheule, Jérôme Montnach, Julien Perrier, Louise C. Lemonnier, Manon Denis, Bertrand Rozec, Benjamin Lauzier, Aurélia A. Leroux

**Affiliations:** 1 Nantes Université, CHU Nantes, CNRS, INSERM, L’institut du Thorax, F-44000 Nantes, France; angelique.blangyletheule.pro@gmail.com (A.B.-L.); jerome.montnach@univ-nantes.fr (J.M.); bertrand.rozec@univ-nantes.fr (B.R.); benjamin.lauzier@univ-nantes.fr (B.L.); 2 Oniris, F-44300 Nantes, France

**Keywords:** equine internal medicine, diagnostic clinical score, retrospective study, sepsis, adult horses

## Abstract

Colic is a leading cause of mortality in horses, particularly when associated with systemic inflammation and organ dysfunction. In human medicine, these complications are associated with sepsis, defined as organ dysfunction caused by a dysregulated, life-threatening host response to infection. However, no consensus sepsis score exists for adult horses. This retrospective study aimed to develop a diagnostic sepsis score for adult horses. Medical records from 295 horses admitted for colic to the Oniris equine emergency department (Nantes, France) between July 2011 and November 2021 were analyzed. Horses were classified as having sepsis or colic based on their final diagnosis. Clinical and laboratory parameters were assessed, and univariate and multivariate logistic regression analyses yielded a predictive score. The optimal threshold was determined by maximizing sensitivity and specificity. Sepsis was diagnosed in 89 horses (30.2%). Six parameters—temperature, heart rate, leukocyte count, red blood cells, creatine kinase, and lactate—were included in the model. The score showed 79% accuracy, with 59% sensitivity and 88% specificity. At a threshold of 3, sensitivity was 52.7%, specificity was 91.6%, and the Youden index was 0.44. This study proposes a rapid, practical sepsis diagnostic score for adult horses with acute gastrointestinal disease, serving as a highly specific tool for confirming sepsis.

## Simple Summary

Colic is a common and serious digestive problem in adult horses that can become life-threatening when it causes widespread inflammation and organ failure. Recognizing when a horse develops a severe infection that leads to sepsis is difficult because no standard method exists. This study aimed to create a simple tool to help veterinarians detect sepsis early in horses with acute digestive problems. By reviewing the medical records of 295 horses admitted for colic over ten years, six key signs were identified, including body temperature, heart rate, blood cell counts, and certain blood chemicals, which together predict the risk of sepsis. These signs were combined into a scoring system that is easy to use and can guide decisions about urgent treatment. Early recognition of sepsis may improve survival, reduce complications, and help veterinarians and owners manage affected horses more effectively.

## 1. Introduction

Equids are particularly susceptible to digestive disorders, including colic [1]. Colic is one of the most prevalent diseases in horses and remains a major cause of morbidity and mortality in this species [2,3]. While medical treatment often proves effective in many cases, gastrointestinal disease can be associated with systemic inflammatory response syndrome (SIRS) and multiple organ dysfunction syndrome (MODS), which may markedly worsen prognosis. In equine medicine, identifying the transition from localized primary injury to systemic compromise remains a major clinical challenge due to the lack of specific diagnostic criteria. In human medicine, such clinical features have been associated with sepsis, which is defined as organ dysfunction caused by a dysregulated, life-threatening host response to infection [4]. The sequential sepsis-related organ failure assessment (SOFA) score, introduced in the Third International Consensus Definitions for Sepsis and Septic Shock (Sepsis-3), has significantly improved the specificity of sepsis diagnosis in human medicine, thus also improving the speed of treatment initiation, the monitoring of patient response to therapy, and outcome prediction [4,5]. Importantly, the SOFA score is designed as an organ dysfunction-based and longitudinal tool to monitor the progression of organ failure over time [4]. However, this scoring system was developed and validated for human patients, and its direct applicability to other species remains uncertain, as it has not been validated across species in veterinary medicine.

No consensus has been established for the definition of sepsis for adult horses, but the veterinary scientific community has agreed to use the same definition as in human medicine [1,6]. Gastrointestinal disorders represent the most frequent etiology of sepsis in adult horses, as the disruption of mucosal integrity facilitates the translocation of bacteria and the absorption of endotoxins into the systemic circulation [1]. Endotoxaemia is a hallmark of this septic process; while comprehensive epidemiological data for the clinical syndrome of sepsis remain limited, circulating endotoxin has been identified in approximately 29% of horses presenting with colic [7]. This underscores the high prevalence of systemic inflammatory challenges in this population and highlights the intrinsic link between intestinal barrier failure and the development of sepsis. Recently, studies have evaluated the use of SIRS and MODS scores in adult horses [8,9], but SIRS lacks specificity and MODS relies on parameters that may be complex or difficult to obtain in routine equine clinical practice. Therefore, there is a critical need for a pragmatic, admission-based tool that utilizes readily accessible variables to identify horses at risk of sepsis upon arrival. An equine equivalent SOFA score seems to be a promising solution to improve the rapid diagnosis and management of septic horses. However, sepsis diagnostic criteria have not yet been clearly validated in horses, and the SOFA score includes physiological parameters that are difficult to measure in routine equine practice (such as mean arterial pressure or inspired oxygen fraction).

The aim of this study is to develop a diagnostic score for sepsis in adult horses with colic. For this purpose, the diagnostic and biological data of horses admitted for colic will be analyzed retrospectively over a 10-year period. We hypothesize that these analyses will highlight a set of independent predictors of sepsis in this population, which can be integrated into a clinically relevant and accurate diagnostic scoring system for the early identification of sepsis in adult horses.

## 2. Materials and Methods

### 2.1. Inclusion Criteria

**Figure 1:**
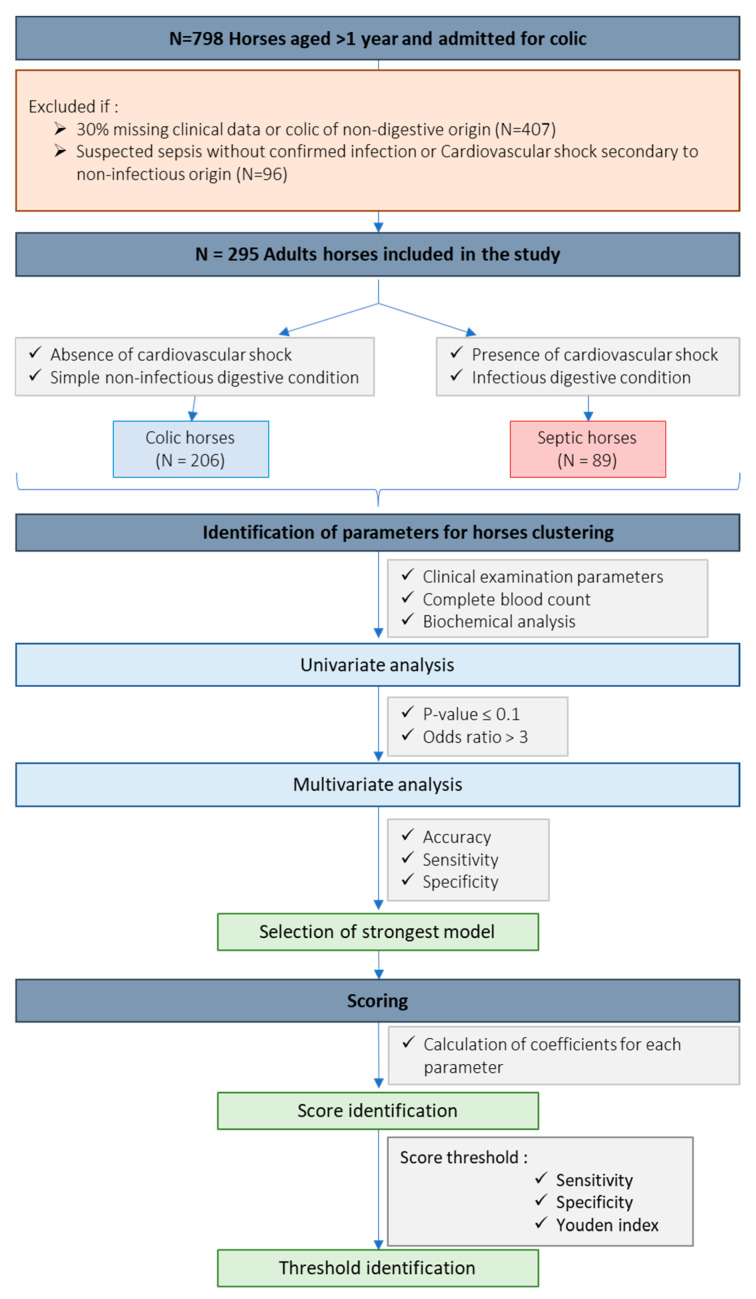
Flow chart of inclusion and exclusion procedures. Horses aged at least one year and admitted for colic to the Oniris VetAgroBio equine emergency department (Nantes, France) were initially included in the study. According to the final diagnosis, horses were divided into 2 groups: (i) horses with colic without signs of sepsis and (ii) horses with colic developing sepsis. Parameters of the admission clinical examination, blood count and biochemical analyses were analyzed using a univariate approach. The parameters selected with the univariate analysis were analyzed by a multivariate analysis. Different multivariate models were performed to select the most discriminating one. A logistic regression was then performed to identify the coefficients of each parameter, resulting in a sepsis diagnostic score.

This study is a retrospective collection of biological and clinical information on cases of horses admitted for colic to the equine teaching hospital of Oniris VetAgroBio Nantes (Nantes, France). Diagnostic test results, diagnoses and outcomes were collected and analyzed retrospectively. A total of 798 adult horses aged more than one year and admitted for colic between July 2011 and November 2021 were included in the study (Figure 1).

### 2.2. Exclusion Criteria

Horses with more than 30% missing clinical data were excluded from the study. Similarly, horses with acute gastrointestinal pathology and suspected sepsis, but no confirmed infection (e.g., infectious diarrhea and septic peritonitis not confirmed by necropsy or bacterial culture), were considered a gray area and excluded from the study. Horses with colic associated with non-infectious systemic causes (e.g., toxicosis) and horses admitted for colic secondary to hepatic or urogenital injuries were excluded. After exclusions, a final cohort of 295 horses was used to develop the score (Figure 1).

### 2.3. Classification of the Horses

The horses were divided into 2 groups, colic (N = 206) and sepsis (N = 89), according to their final diagnosis (Figure 1). The final diagnosis was made by necropsy, surgery or following a thorough diagnostic evaluation. Horses were classified into the “colic” or “sepsis” groups based on their final diagnosis. This diagnosis was independently established in a double-blinded manner by a European Veterinary Specialist in Equine Internal Medicine (AAL, Dip. ECEIM) to avoid bias related to individual clinical or laboratory parameters. Such a methodological approach aligns with practices in human medical research, where the expert-validated final diagnosis is used as the reference standard to minimize selection bias [10,11,12]. Horses were classified in the colic group if they were diagnosed with simple, uncomplicated gastrointestinal disease without evidence of cardiovascular shock, systemic infection or ischemic intestinal injury. This group included horses with conditions such as colon displacement, small or large intestinal simple obstruction, chronic inflammatory bowel diseases and gastric ulceration and/or impaction. Horses were classified in the sepsis group if they were diagnosed with septic peritonitis confirmed by cytology, biochemical analysis, bacterial culture, or necropsy findings, in association with advanced ischemic intestinal injury, intestinal rupture, or idiopathic peritonitis. Horses with colic and confirmed infectious diarrhea (caused by *Salmonella* spp., *Clostridium perfringens* or *Clostridium difficile*) were also included. All horses classified in the sepsis group exhibited clinical evidence of cardiovascular shock recorded in the medical file and verified by the specialist. Cardiovascular shock was defined by the presence of one or more clinical and laboratory abnormalities consistent with poor peripheral perfusion, including cold extremities, abnormal mucous membrane color, prolonged capillary refill time (CRT), tachycardia, and/or hyperlactatemia [13].

### 2.4. Clinical and Laboratory Variables Assessed at Admission

A retrospective study of the admission examinations from case reports was performed, and a non-selective collection of all available routine parameters was conducted to ensure an objective baseline for analysis. Clinical data were collected, including heart rate, respiratory rate, CRT, presence of reflux after nasogastric intubation, and observation of ileus on abdominal ultrasound. Collected laboratory data included a complete blood count, including leukocytes, neutrophils, lymphocytes, monocytes, red blood cells, hematocrit, hemoglobin concentration, and platelets, and a biochemical analysis, including urea, creatinine, alkaline phosphatases, aspartate aminotransferase, albumin, total proteins, creatine kinase, gamma glutamyl transferase, total bilirubin and fibrinogen.

### 2.5. Identification of Parameters for Horses Clustering

Parameters that are easily accessible and commonly used in clinical practice were included in the study. Each parameter was classified as physiological or pathological according to the laboratory standards detailed in Table S1. Cases with excessive missing data were handled according to the exclusion criteria described in Section 2.2. For the remaining cases, missing values were treated as unavailable observations and were not included in the corresponding statistical analyses. The univariate analysis was used to calculate an odds ratio (OR), 95% confidence intervals (95% CI) and a *p*-value for each parameter. An OR > 1 corresponds to a positive association with sepsis, whereas a ratio < 1 corresponds to a negative association. Parameters with a *p*-value less than 0.1 and an odds ratio greater than 3 were selected for the multivariate analysis (Figure 1). These thresholds were predefined prior to model construction in order to retain variables showing both statistical association and a clinically meaningful strength of effect, while limiting the number of candidate predictors and reducing the risk of model overfitting.

Then, the selected parameters were analyzed using multivariate analysis, with the number of parameters limited to a maximum of eight to maintain clinical practicality and model parsimony. This also ensures statistical stability by respecting a sufficient events-per-variable ratio and facilitates the clinical applicability of the score. Prediction models intended for routine clinical use should rely on a limited number of predictors to improve interpretability and facilitate implementation in clinical practice [14]. An iterative procedure was used to optimize the combination of parameters that best discriminated septic horses from colic horses. During this iterative process, when several variables reflected the same biological function, the parameter that contributed the most consistently to the model’s discriminative performance was retained. To study the strength of the model in predicting sepsis, 70% of patients in the cohort were randomly selected using stratified random sampling (to maintain disease prevalence across subsets) to train the generalized linear model (GLM) regression, which was then validated on the remaining 30% of patients. Accuracy, sensitivity and specificity values were calculated and used to select the most discriminating model (Figure 1).

### 2.6. Scoring

To facilitate the clinical use of the selected model, a diagnostic score was established. Logistic regression was employed to assign a coefficient to each variable based on its contribution in the multivariate analysis. If a parameter’s value fell outside the physiologically defined thresholds, it contributed to the score by adding the coefficient value associated with that parameter. The diagnostic score was calculated as the sum of all the coefficients (Figure 1).

Then, the threshold value of the score was investigated. Sensitivity and specificity values were calculated for each possible threshold and the diagnostic performance of each was evaluated using the Youden index. This index, which ranges from 0 to 1, provides a global measure of test effectiveness, with a value of 1 indicating perfect discrimination between septic and non-septic cases, and a value of 0 indicating no discriminative ability (Figure 1).

### 2.7. Statistical Analysis

The numerical and categorical variables of the clinical data of patients were respectively analyzed with a Student test and a Chi^2^ test using the “*tableby*” function of the R package “arsenal”, after verifying normality of continuous variables with the Shapiro–Wilk test and equality of variances with Fisher’s F-test. Univariate and multivariate analyses were carried out using the R software “caret” and their graphic representation was carried out with the ‘*finalfit’* function of the R package “*forestmodel*”. A value of *p* < 0.05 was considered significant. All analyses were performed using R software (version 4.2.0).

## 3. Results

### 3.1. Cohort Characterization

Demographic data were collected and provided in Table 1 and Table S2. Intergroup comparison of demographic parameters did not reveal differences in sex distribution (*p* = 0.75) or age between colic and septic horses (colic: 10.40 ± 6.52 years vs. sepsis: 12.12 ± 8.17 years, *p* = 0.06). Regarding breed distribution (*p* < 0.01), a higher proportion of ponies was observed in the sepsis group (44.0% vs. 17.8%), while warmbloods were more frequently represented in the colic group (65.8% vs. 36.9%). No significant differences were observed for other breed types (Table 1).

Clinic data were collected and provided in Table 2 and Table S2. Concerning clinical examination parameters, nasogastric reflux and/or ileus, temperature, heart rate, respiratory rate, and CRT were significantly higher in the sepsis group.

Regarding blood cell counts, leukocyte and neutrophil counts were significantly lower in the sepsis group compared to the colic group. No significant difference was observed for lymphocyte and platelet counts. Monocyte count, hematocrit, hemoglobin concentration, and red blood cell count were significantly higher in the sepsis group (Table 2).

In terms of biochemical parameters, creatinine, urea, alkaline phosphatase, creatine kinase, aspartate aminotransferase, and lactate concentrations were significantly higher in the sepsis group compared to the colic group, whereas total protein and albumin concentrations were significantly lower. No significant differences were observed for total bilirubin and gamma-glutamyl transferase (GGT) levels (Table 2).

### 3.2. Identification of Parameters for Horses Clustering

Univariate analysis was conducted to identify parameters associated with an increased risk of sepsis (Table 3). Among clinical parameters, abnormal body temperature (<36 or >38.5 °C) was significantly associated with sepsis (OR = 17.62 [95% CI = 6.27–59.92], *p* < 0.001). For hematological parameters, abnormal red blood cell count (RBC) (<5.5 or >12.5 cells/µL) was a strong predictor of sepsis (OR = 35.62 [95% CI = 6.99–650.89], *p* = 0.001), this finding was present in 21.3% (19/89) of septic horses compared to 1% (2/206) of horses in the colic group (Table S2). Additionally, an elevated monocyte count (>1000 cells/µL) was also significantly associated with sepsis (OR = 9.23 [95% CI = 2.24–62.28], *p* = 0.006). Among biochemical parameters, increased creatinine concentration was significantly associated with sepsis (OR = 8.94 [95% CI = 2.71–40.29], *p* = 0.001) (Table 3).

**Figure 2:**
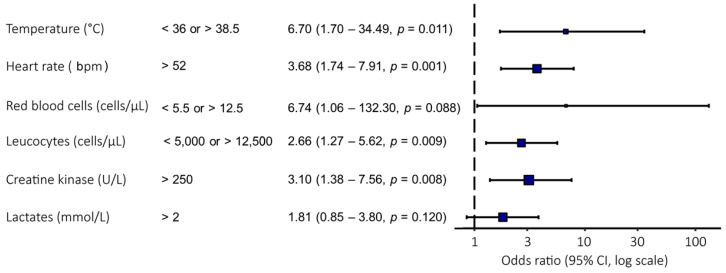
Description of the strongest multivariate analysis model identified in the study.

Variables identified as significant in univariate analysis were subsequently entered into multivariate models to determine their combined predictive value (Figure S1). A forest plot summarized the odds ratios for a model including temperature, heart rate, red blood cell count, leukocyte count, creatine kinase, and lactate (Figure 2). While the model as a whole demonstrated high diagnostic performance (accuracy: 0.79; sensitivity: 59%; specificity: 88%), not all individual variables remained independent predictors. Specifically, temperature, heart rate, leukocyte count, and creatine kinase maintained statistical significance, whereas red blood cell count (*p* = 0.088) and lactate (*p* = 0.120) did not reach the significance threshold in this multivariate context (Figure 2).

### 3.3. Implementation of a Sepsis Diagnostic Score

To facilitate clinical implementation, a diagnostic score for sepsis was developed based on the multivariate model. A score was attributed to each parameter based on its relative weight in the sepsis classification (Table 4). The value of the diagnostic score was obtained by summing the individual coefficients associated with each parameter.

To achieve high specificity in diagnosing sepsis, a threshold score of 3 is recommended, corresponding to a sensitivity of 52.69% and a specificity of 91.58%, and a positive predictive value (PPV) of 74.2%. In clinical practice, using a threshold of 2 may be more appropriate to maximize case detection, offering a higher sensitivity (77.42%) and negative predictive value (NPV = 89.9%), but at the expense of a lower specificity (75.25%) and PPV (54.1%) (Table 5). According to the Youden index, the optimal performance of the diagnostic test is obtained at a threshold of 2. However, a threshold of 3 provides greater diagnostic confidence by significantly increasing the PPV, reducing the risk of false positives. Notably, a score of 2 can be reached with a single strongly predictive parameter (e.g., abnormal temperature or leukocyte count), whereas a score of 3 requires the combination of at least two parameters, thereby increasing diagnostic confidence.

## 4. Discussion

Acute gastrointestinal disorders are a major clinical concern in horses. Sepsis, which is associated with increased morbidity and mortality, represents the most frequent and severe complication in equine clinics. It is essential to improve the diagnosis of equine patients with sepsis in order to implement an appropriate therapeutic strategy to limit morbidity and mortality. In this context, the development of a diagnostic score for sepsis in adult horses is of considerable clinical value. To our knowledge, this is the first study to provide a cost-effective and user-friendly scoring system for the diagnosis of sepsis in adult horses, relying exclusively on parameters that are readily accessible in routine veterinary practice.

We identified a combination of six parameters, including temperature, heart rate, leukocytes, red blood cells, creatine kinase, and blood lactate levels, as an effective combination for sepsis diagnosis. Univariate analysis demonstrated that each of these parameters was significantly associated with sepsis (*p* < 0.05). However, in the multivariate model, only a subset remained independent predictors. This highlights the complex interaction between clinical and laboratory indicators in outcome prediction, as previously observed by Spadari and his collaborators in the context of post-operative colic prognosis [15]. This multivariate approach follows the logic that systemic compromise is best captured by an integrated model of physiological variables, a concept further supported by recent findings suggesting that even behavioral indicators reflect overall systemic homeostasis [16].

The six parameters identified in our score are routinely assessed by equine veterinarians and have already been individually proposed to highlight either SIRS or multi-organ dysfunction. Notably, blood lactate was included in the first definition of equine sepsis [17] while temperature, heart rate, and leukocytes have recently been incorporated into a SIRS scoring system [9]. Creatine kinase has been used as a marker of musculoskeletal injury or dysfunction [8]. However, our study is the first to combine these parameters into a composite diagnostic score, improving accuracy for sepsis detection.

It is noteworthy that, in addition to lactate levels or leukocyte counts, elevated CK was uniquely associated with sepsis in our dataset compared to other studies [8,9]. Elevated CK levels have also been reported in human sepsis complicated by rhabdomyolysis [18,19], suggesting possible musculoskeletal or cardiac involvement. Since total CK reflects contributions from multiple isoenzymes, including those from skeletal muscle, heart, and the central nervous system, the precise origin of the elevation remains unclear. Although a central origin is unlikely in the absence of significant neurological signs, myocardial involvement cannot be ruled out, especially since myocardial dysfunction has been reported in severe equine SIRS cases [17]. Elevated CK concentrations in horses with colic are often multifactorial and require careful clinical interpretation. While primary muscle damage or myocardial involvement are possible explanations, CK activity may also increase as a consequence of transport-induced stress [20,21]. In the present study, horses were admitted following relatively short journeys from the local area; therefore, long-distance transport was unlikely to account for the observed CK elevations. Moreover, CK elevation may reflect the severity of clinical symptoms, such as prolonged recumbency or prolonged struggling [22]. In addition, increasing evidence suggests that CK concentrations may be associated with the severity of the underlying disease process. In particular, CK has been identified as a potential biomarker for strangulating small intestinal lesions, which are commonly associated with ischaemia and systemic inflammatory responses [23]. Therefore, in our population, the observed CK concentrations likely reflect a combination of secondary muscular effects related to severe colic and the severity of the primary intestinal injury

In light of these results, it is relevant to compare our approach with the SOFA score commonly used in human medicine to assess organ dysfunction progression. The SOFA score evaluates six organ systems (respiratory, cardiovascular, hepatic, coagulation, renal and neurological), assigning a severity score from 0 to 4 to each. Surprisingly, our multivariate analysis did not identify the typical markers of multi-organ dysfunction usually observed in sepsis, such as renal, hepatic, or pulmonary dysfunction, but instead highlighted parameters reflecting cardiovascular, musculoskeletal, and inflammatory impairments. These findings are in accordance with the MODS SGI score for horses with acute surgical colic proposed by McConachie and collaborators, which similarly identified cardiovascular and musculoskeletal dysfunctions [8]. However, unlike our study, the MODS SGI score also incorporates markers of renal, hepatic, respiratory, coagulation, and gastrointestinal function, similarly to SOFA. This discrepancy may result from differences in assessment timing, since their assessments occurred post-surgery, possibly reflecting more advanced stages of MODS. However, it is important to clarify that our score is designed as an admission tool for rapid risk identification rather than a longitudinal measure of organ dysfunction progression. While it mimics the SOFA score’s logic by integrating multiple organ systems, future longitudinal studies are required to evaluate its utility in monitoring the progression of MODS in septic horses over time.

These findings highlight the urgent need for an accurate and reliable diagnostic tool for equine sepsis. The model developed in this study effectively identifies septic horses, with reasonable sensitivity (59%) and high specificity (88%). To facilitate practical use by veterinarians, we derived a diagnostic score with an optimal threshold. In our cohort of adult horses, a threshold between 2 and 3 was evaluated: a cut-off of 2 yields higher sensitivity (77.4%) but lower specificity, while a cut-off of 3 achieves higher specificity (91.6%) but reduced sensitivity (52.7%). The Youden index supports the better overall discriminative power of the threshold at 2. However, a score of 2 can be reached using a single parameter, which may reduce specificity in clinical practice. From a clinical perspective, prioritizing higher specificity may be preferable to minimize false-positive diagnoses. For these reasons, a threshold of 3 was prioritized. The absence of significant differences in demographic variables such as age and sex between groups suggests that the score may remain robust across a diverse adult horse population.

Furthermore, our cohort consisted of horses with no comorbidities. However, in a clinical context, sepsis must be diagnosed amidst populations potentially affected by other conditions that may alter score parameters—especially temperature. Given this context, a threshold of 2 may be appropriate only if fewer than two parameters are abnormal; otherwise, a threshold of 3 is currently more suitable. Importantly, these threshold values need to be validated in prospective cohorts to confirm their clinical utility.

To our knowledge, no comparable diagnostic score has been developed for adult horses, although several sepsis scores for foals have been studied extensively. These foal sepsis scores report sensitivities and specificities ranging from 56.4% to 87.5% and 76% to 92.8%, respectively [24,25,26,27]. Similarly to these scores, the moderate sensitivity of our adult horse score may reflect the continuum between gastrointestinal infection and sepsis [28]. Notably, the widely used foal sepsis score by Wong and collaborators achieves a similar sensitivity (62%) but lower specificity (64%) compared to our adult horses score [27]. Therefore, our score, with a threshold of 3, could be implemented in clinical practice to support sepsis diagnosis, albeit cautiously and in conjunction with thorough clinical evaluation. In addition, improving this score with emerging protein biomarkers in the future may enhance its sensitivity and overall diagnostic performance.

The scoring model developed in this study is based on a highly refined design and is readily applicable in routine clinical practice. However, several limitations must be acknowledged.

First, the model was based on a monocentric cohort with a relatively limited number of septic horses. Indeed, there is ongoing discussion regarding how geographical location may impact the reproducibility of the results [27]. Furthermore, the exclusion of horses with an uncertain phenotype, where sepsis could neither be confirmed nor ruled out, may have introduced a spectrum bias, potentially inflating the observed diagnostic accuracy. To address these concerns, a prospective validation involving a larger, multicentric and multinational cohort including all phenotypes of colic cases is essential. This validation would not only improve the statistical power of the model but also assess its robustness across different populations and clinical settings. Second, assessing the ability of the score to distinguish sepsis from other critical non-septic conditions would be particularly relevant in a clinical context, where overlapping clinical signs often complicate decision-making. Future studies could include comparisons with cardiovascular shock secondary to other causes such as hemorrhage. Another limitation involves potential confounding factors such as pre-admission treatments. While data were collected at the time of arrival, prior administration of non-steroidal anti-inflammatory drugs by field veterinarians could influence parameters like heart rate or temperature. Similarly, while our cohort focused on primary gastrointestinal sepsis, the presence of unidentified comorbidities in a general population could reduce the score’s specificity, particularly for non-specific markers like temperature or heart rate. Additionally, it would be valuable to test the score prospectively in a general admission cohort with systematic blood cultures to better evaluate its diagnostic performance. Lastly, the scope of septic etiologies evaluated in this study was limited to horses presenting with gastrointestinal disease, specifically colic complicated by sepsis. Although gastrointestinal pathologies remain one of the main causes of sepsis, testing the application of this score to other common aetiologies, such as metritis or pneumonia, is necessary to determine its broader clinical utility. Moreover, while our cohort was selected to minimize comorbidities, the presence of concurrent diseases in a more heterogeneous clinical population could influence the scoring system’s specificity.

## 5. Conclusions

In conclusion, this study presents for the first time a diagnostic sepsis score in adult horses. The score is based on six parameters (temperature, heart rate, red blood cells, leukocytes, creatine kinase and blood lactates) that are readily accessible in clinical practice. A score greater than or equal to 2 offers a good balance between sensitivity and specificity, while a score greater than or equal to 3 increases specificity at the expense of sensitivity. This score has the potential to serve two purposes: (i) to potentially assist clinicians in identifying patients who may benefit from ad hoc therapeutic strategies to limit mortality associated with sepsis, and (ii) to enable a more accurate assessment of sepsis incidence in adult horses. A blank, user-friendly version of the score is provided in the Supplementary Materials to facilitate its application in clinical settings (Table S3).

## Figures and Tables

**Table 1:** Demographic characteristics of horses included in the study.

	Colic (N = 206)	Sepsis (N = 89)	*p* Value
Gender			0.75
Stallion	17 (8.3%)	10 (11.2%)	
Gelding	94 (45.6%)	40 (44.9%)	
Mare	95 (46.1%)	39 (43.8%)	
Age (years)	10.40 ± 6.52	12.12 ± 8.17	0.06
Breed type			<0.01
Draft	3 (1.5%)	5 (6.0%)	
Pony	36 (17.8%)	37 (44.0%)	
Standardbred	11 (5.4%)	5 (6.0%)	
Thoroughbred	19 (9.4%)	6 (7.1%)	
Warmblood	133 (65.8%)	31 (36.9%)	

The numerical values are expressed as the number of events, percentages or mean ± standard deviation.

**Table 2:** Clinical and hematobiochemical data of horses included in the study.

	Colic (N = 206)	Sepsis (N = 89)	*p* Value
Clinical examination			
Nasogastric reflux and/or ileus (yes)	71 (34.5%)	64 (71.9%)	<0.01
Rectal temperature (°C)	37.52 ± 0.54	37.89 ± 0.95	<0.01
Heart rate (bpm)	47.98 ± 12.80	72.85 ± 23.90	<0.01
Respiratory rate (mpm)	20.14 ± 8.55	28.03 ± 12.82	<0.01
Capillary refill time (s)	1.93 ± 0.77	2.73 ± 1.02	<0.01
Complete blood count			
Leukocytes (×10^3^/µL)	10.77 ± 3.57	9.67 ± 5.39	0.04
Neutrophils (×10^3^/µL)	7.96 ± 3.31	6.40 ± 4.81	<0.01
Lymphocytes (×10^3^/µL)	2.23 ± 0.97	2.27 ± 1.25	0.96
Monocytes (×10^3^/µL)	0.43 ± 0.22	0.61 ± 0.80	<0.01
Hematocrit (%)	35.03 ± 6.40	46.43 ± 13.45	<0.01
Hemoglobin (g/dL)	12.68 ± 2.11	16.21 ± 4.19	<0.01
Red Blood Cells (×10^6^/µL)	7.69 ± 1.40	9.79 ± 2.60	<0.01
Platelets (×10^3^/µL)	146.60 ± 56.01	134.64 ± 55.17	0.10
Biochemical analysis			
Total protein (g/dL)	67.27 ± 7.06	62.87 ± 10.35	<0.01
Albumin (g/dL)	29.16 ± 3.94	27.52 ± 5.58	<0.01
Creatinine (mg/dL)	12.50 ± 4.52	15.62 ± 12.76	<0.01
Urea (g/L)	0.34 ± 0.12	0.46 ± 0.22	<0.01
Alkaline phosphatase (U/L)	274.92 ± 246.77	394.45 ± 424.38	<0.01
Gamma-glutamyl transferase (U/L)	45.39 ± 52.37	57.47 ± 85.25	0.16
Total bilirubin (mg/dL)	24.72 ± 16.02	26.29 ± 17.96	0.53
Creatine kinase (U/L)	380.68 ± 452.29	1070.76 ± 2059.34	<0.01
Aspartate aminotransferase (U/L)	371.89 ± 180.26	468.47 ± 356.87	<0.01
Lactates (mmol/L)	1.89 ± 1.56	5.42 ± 4.77	<0.01

bpm: beats per minute; mpm: movements per minute; sec: seconds. The numerical values were expressed as mean ± standard deviation.

**Table 3:** Univariate analysis of risk factors of sepsis.

Parameters Evaluated for Sepsis Outcome	OR	95% CI	*p*-Value
Age (years)	1.84	1.04–3.20	0.033
Nasogastric reflux and/or ileus	4.71	2.79–8.14	<0.001
Rectal temperature (°C)	17.06	6.27–59.92	<0.001
Heart rate (bpm/min)	8.74	5.05–15.52	<0.001
Respiratory rate (mpm/min)	5.14	2.97–9.04	<0.001
Capillary refill time (s)	3.93	2.34–6.68	<0.001
Leukocytes (cells/µL)	3.14	1.87–5.30	<0.001
Neutrophils (cells/µL)	1.57	0.96–2.59	0.074
Lymphocytes (cells/µL)	1.53	0.88–2.64	0.130
Monocytes (cells/µL)	9.23	2.24–62.28	0.006
Hematocrit (%)	21.44	10.22–49.73	<0.001
Hemoglobin (g/dL)	23.10	9.39–69.79	<0.001
Red blood Cells (cells/µL)	35.62	6.99–650.89	0.001
Platelets (×10^6^/µL)	1.82	0.93–3.50	0.074
Total protein (g/dL)	3.47	1.99–6.07	<0.001
Albumin (g/dL)	3.02	1.66–5.52	<0.001
Creatinine (mg/dL)	8.94	2.71–40.29	0.001
Uremia (g/dL)	5.32	2.59–11.40	<0.001
Alkaline phosphatase (U/L)	2.85	1.61–5.08	<0.001
Gamma-glutamyl transferase (U/L)	0.68	0.40–1.17	0.164
Total bilirubin (mg/dL)	2.49	1.17–5.33	0.018
Creatine Kinase (U/L)	3.75	2.04–7.28	<0.001
Aspartate aminotransferase (U/L)	2.49	1.44–4.30	0.001
Lactates (mmol/L)	5.94	3.44–10.52	<0.001

bpm: beats per minute; mpm: movements per minute; sec: seconds; OR: odds ratio; CI: confidence interval.

**Table 4:** Summary of the adult horse sepsis diagnostic score.

Parameter	Threshold	Score
Rectal temperature (°C)	[36–38.5]	0
	<36 or >38.5	2
Heart rate (bpm/min)	≤52	0
	>52	1
Leukocytes (cells/µL)	[5000–12,500]	0
	<5000 or >12,500	2
Red blood Cells (cells/µL)	[5.5–12.5]	0
	<5.5 or >12.5	1
Creatine Kinase (U/L)	≤250	0
	>250	1
Lactates (mmol/L)	≤2	0
	>2	1

**Table 5:** Identification of the threshold value of the sepsis diagnostic score.

Score	0	1	2	3	4	5	6	7
Sensitivity (%)	100.00	96.77	77.42	52.69	25.81	20.43	8.60	4.30
Specificity (%)	0.00	33.66	75.25	91.58	98.02	99.01	100.00	100.00
VPP (%)	31.5	40.2	54.1	74.2	85.7	90.5	100	100
VPN (%)	-	95.8	89.9	81.1	74.1	73.0	70.4	69.4
Youden Index	0	0.3	0.53	0.44	0.19	0.19	0.09	0.04

## Data Availability

The data supporting the findings of this study are available from the corresponding author upon reasonable request.

## Supplementary Materials

The following supporting information can be downloaded at https://www.mdpi.com/article/10.3390/ani16060962/s1: Supplemental Table S1: Description of threshold values for biological parameters used in the study. Supplemental Table S2: Detailed information about patient cohorts, demographic and clinical routine parameters. Supplemental Table S3: Sepsis diagnostic score template for clinical use. This table is provided to facilitate clinical application by veterinarians, allowing them to print and complete the score during patient assessment. Supplemental Figure S1: Description of the different multivariate analysis models performed.
