# Supplementary material for: New Diagnostic Score for Sepsis in Adult Horses with Acute Gastrointestinal Disease
Source: Animals (Basel). 2026 Mar 19;16(6):962. doi: 10.3390/ani16060962 (PMC13023294; doi:10.3390/ani16060962)
Supplement: Supplementary file 1 [file animals-16-00962-s001.zip › Table S3 - Clinically usable score.pdf]

Parameter	Threshold	Score
Rectal temperature (°C)	[36 - 38.5]	0
	< 36 or > 38.5	2
Heart rate (bpm/min)	≤ 52	0
	> 52	1
Leucocytes (cells/µL)	[5000 - 12500]	0
	< 5000 or > 12500	2
Red blood Cells (cells/µL)	[5.5 – 12.5]	0
	< 5.5 or > 12.5	1
Creatine Kinase (U/L)	≤ 250	0
	> 250	1
Lactates (mmol/L)	≤ 2	0
	> 2	1
**Total score**		
