# Supplementary figures and images for: New Diagnostic Score for Sepsis in Adult Horses with Acute Gastrointestinal Disease
Source: Animals (Basel). 2026 Mar 19;16(6):962. doi: 10.3390/ani16060962 (PMC13023294; doi:10.3390/ani16060962)

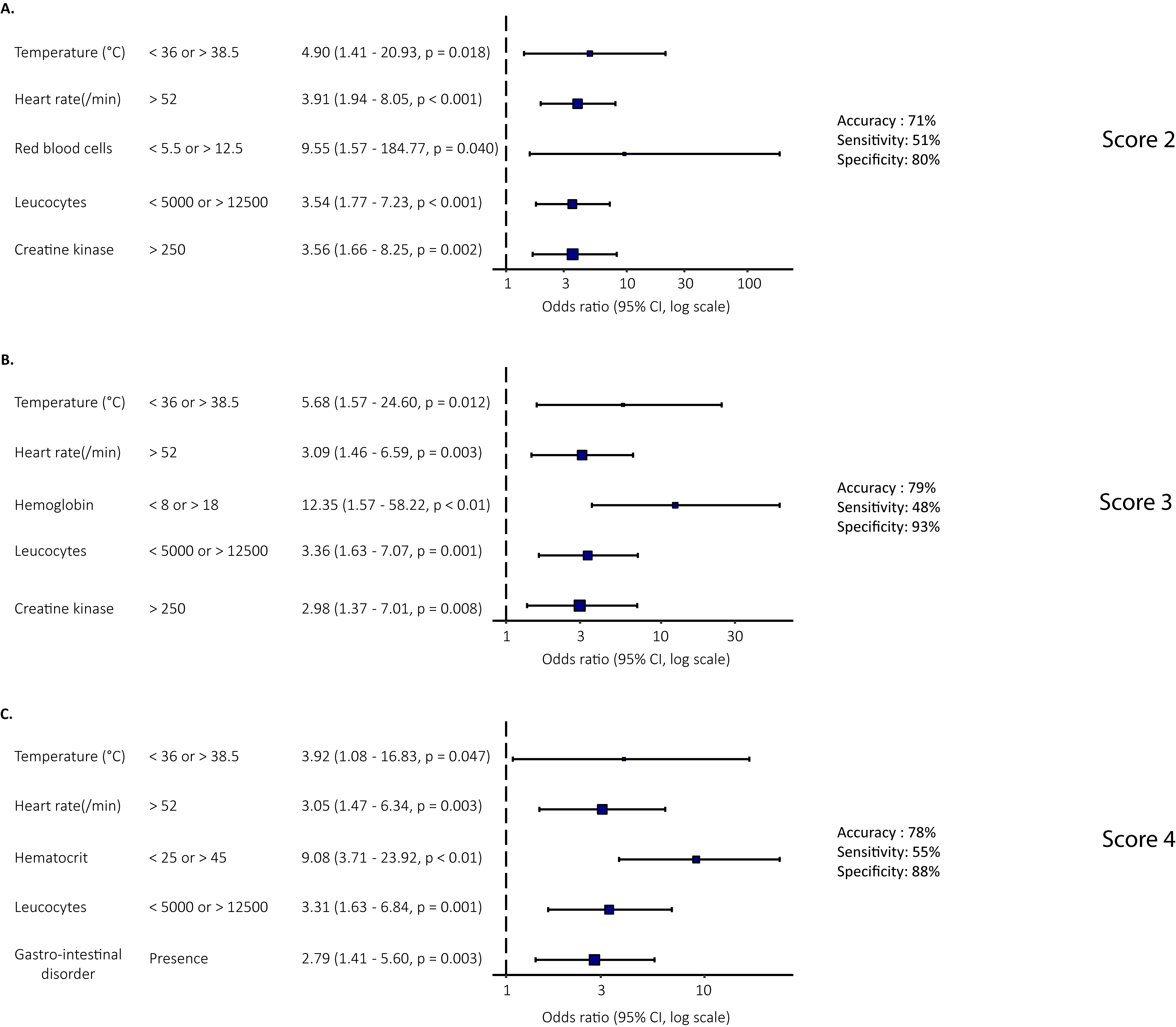

Supplement: Supplementary file 1 [file animals-16-00962-s001.zip › Figure S1 Description of the different multivariate analysis models performed.tif]
